# Design and Fabrication of Interdigital Nanocapacitors Coated with HfO_2_

**DOI:** 10.3390/s150101998

**Published:** 2015-01-16

**Authors:** Gabriel González, Eleazar Samuel Kolosovas-Machuca, Edgar López-Luna, Heber Hernández-Arriaga, Francisco Javier González

**Affiliations:** 1 Cátedras Conacyt, Universidad Autónoma de San Luis Potosí, 78000 San Luis Potosí, Mexico; E-Mail: gabriel.gonzalez@uaslp.mx; 2 Coordinación para la Innovación y la Aplicación de la Ciencia y la Tecnología, Universidad Autónoma de San Luis Potosí, 78000 San Luis Potosí, Mexico; E-Mails: samuel.kolosovas@uaslp.mx (E.S.K.-M.); edgar.luna@uaslp.mx (E.L.-L.); heber.hernandez@uaslp.mx (H.H.-A.)

**Keywords:** interdigital nanocapacitors, finite element method, capacitance

## Abstract

In this article nickel interdigital capacitors were fabricated on top of silicon substrates. The capacitance of the interdigital capacitor was optimized by coating the electrodes with a 60 nm layer of HfO_2_. An analytical solution of the capacitance was compared to electromagnetic simulations using COMSOL and with experimental measurements. Results show that modeling interdigital capacitors using Finite Element Method software such as COMSOL is effective in the design and electrical characterization of these transducers.

## 1. Introduction

**Figure 1.**
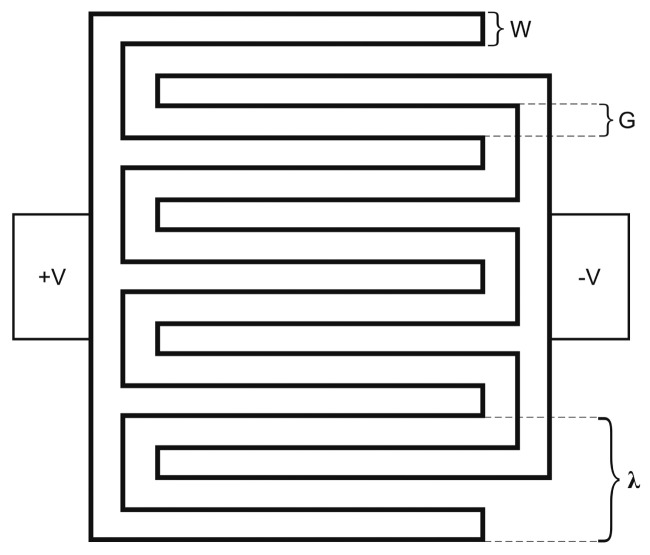
Geometry of an interdigital capacitor.

Interdigital capacitors (IDCs) are one of the most used transducers in chemical and biological sensors where a change in capacitance or impedance is measured as a response to the interaction between the analyte and a sensitive layer [1]. Interdigital capacitors are used also for the evaluation of near-surface electrical properties, such as conductivity, permeability, and permittivity of materials, and for improving the *Q*-factor in resonators [2,3]. The basic geometry of an IDC is defined by the parameters shown in Figure 1. These parameters include the number of electrodes *N*, electrode width *W*, electrode length *L* and the separation between electrodes *G*. The total capacitance of the IDC depends on these parameters and on the characteristics of the substrate where the IDC is mounted on. In addition, the thickness of the electrode and its conductivity will also come into play in the capacitance of the IDC [4,5].

Typical microelectronic fabrication processes based on silicon substrates are being used for the miniaturization of the IDCs. It is very well known that the capacitance is proportional to the electric permittivity such that a dielectric material with higher permittivity constant is required to increase the capacitance [6]. Silicon dioxide has been used as the primary dielectric material for silicon-based devices for many years. Alternatives for replacing SiO_2_ by dielectrics with high permittivity constants are currently being investigated [7–9]. More recently, many dielectrics with high permittivity constants have been suggested as replacement candidates for silicon dioxide. Among these materials with high dielectric constant and good compatibility with silicon we find hafnium dioxide (HfO_2_) [10]. Hafnium dioxide is considered as one of the best dielectric materials for IDCs due to its high thermodynamic stability on silicon and high electric permittivity compared with other dielectrics (*ϵ_r_* ≈ 25).

In this article we designed and fabricated a nickel IDC on a silicon substrate and deposited a 60 nm layer of HfO_2_ to optimize its capacitance. We compare the analytical solution and the numerical simulations using COMSOL with experimental measurements. Our results show good agreement between the numerical simulations and experimental measurements.

The article is organized as follows. We first start by using the analytical model for IDCs to calculate the capacitance of our device. We then apply numerical simulations to obtain the electrostatic potential and capacitance of our IDCs. We subsequently present our experimental results for the IDCs and compare them with the analytical and numerical results.

## 2. Analytical Model

Several theoretical models for IDCs have been developed based on conformal mapping and partial capacitance technique to derive closed-form expressions of the capacitance for the devices.

**Figure 2.**
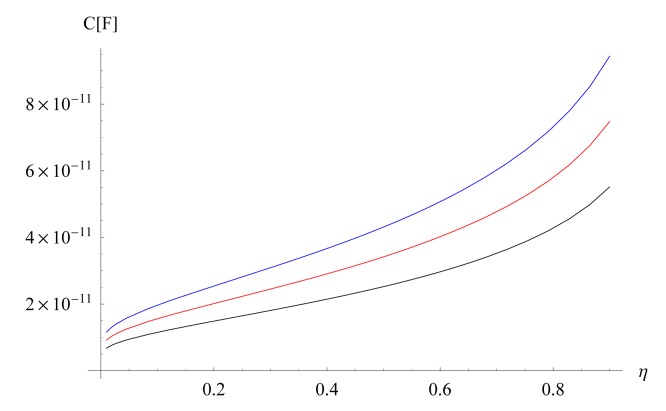
Capacitance as a function of *η* for *N* = 32 (Black), *N* = 40 (Red) and *N* = 50 (Blue). We used *ϵ*_0_ = 8.8 × 10^−12^ F/m, *L* = 8 × 10^−3^ m, *ϵ_S_* = 11.7 F/m, *ϵ*_1_ = 1 y *λ* = 400 × 10^−6^ m.

The conformal mapping technique allows us to analyze the electrical potential distribution inside the IDC in the same way as in a parallel plate capacitor but in a new coordinate system. The closed-form solution of the total capacitance between the negative and the positive electrodes of the IDC is equal to [11]

(1) $$\begin{array}{ll} C_{\text{total}}=\left(N-3\right)\frac{C_{I}}{2}+2\frac{C_{I}C_{E}}{C_{I}+C_{E}}, & \text{for}N>3 \end{array}$$

where *N* represents the number of electrodes, and *C_I_* and *C_e_* are given by:

$$\begin{array}{cc} C_{I} & =\epsilon_{0}L\left(\frac{K(k_{I\infty })}{K({k^{\prime }}_{I\infty })}+(\epsilon_{1}-1)\frac{K(k_{I,1})}{K({k^{\prime }}_{I,1})}+\epsilon_{S}\frac{K(k_{I\infty })}{K({k^{\prime }}_{I\infty })}\right)\\ C_{E} & =\epsilon_{0}L\left(\frac{K(k_{E\infty })}{K({k^{\prime }}_{E\infty })}+(\epsilon_{1}-1)\frac{K(k_{E,1})}{K({k^{\prime }}_{E,1})}+\epsilon_{S}\frac{K(k_{E\infty })}{K({k^{\prime }}_{E\infty })}\right) \end{array}$$

where *K*(*k*) is the complete elliptic integral of first kind with modulus *k* and complementary modulus $k^{\prime }=\sqrt{1-k^{2}}$, $k_{I\infty }=\sin\left(\frac{\pi \eta }{2}\right)$, $k_{E\infty }=\frac{2\sqrt{\eta }}{1+\eta }$, *η* = 2*W*/*λ, λ* = 2(*W* + *G*), *ϵ*_0_ is the electric permittivity of free space, *ϵ*_1_ is the relative electric permittivity of the top layer and *ϵ_S_* is the relative electric permittivity of the substrate. In Figure 2, the dependence of the capacitance as a function of *η* is shown for the case of the IDCs on a silicon substrate.

Figure 2 clearly shows that the enhanced capacitance as the number of electrodes increases.

## 3. Numerical Simulations

**Figure 3.**
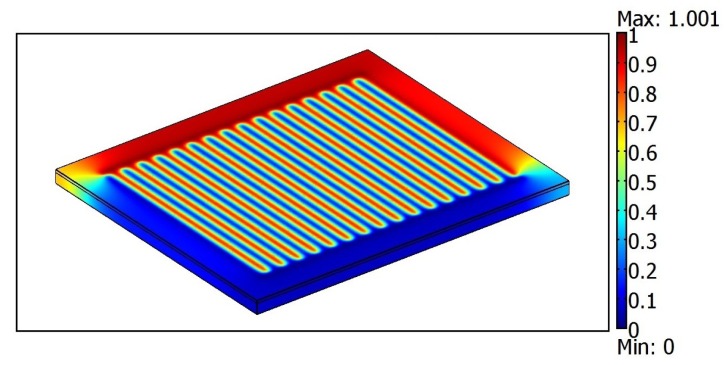
The 3D simulation of the electrostatic potential for an Interdigital capacitor. We used *ϵ*_0_ = 8.8 × 10^−12^ F/m, *L* = 8 × 10^−3^ m, *ϵ_S_* = 11.7 F/m, *ϵ*_1_ = 1 y *λ* = 400 × 10^−6^ m.

The numerical simulation of the IDC was performed by using the software package COMSOL Multiphysics (version 3.5a), which provides a good multi-physics platform for 3D modeling. COMSOL Multiphysics is a software package widely used for modeling. This software not only helps to define the geometry but also helps to visualize the end results. The results of the simulations help us to design the IDC with optimal dimensions and capacitance for practical applications. To perform simulations, we used software COMSOL Multiphysics and its MEMS module, “Electrostatics”, which allowed us to make 3D design of sensors structure and to obtain value of capacitance of the simulated structure. In Figure 3 we have the 3D numerical simulation of the electrostatic potential for IDC with *N* = 32 and when a voltage of 50 mV is applied.

**Figure 4.**
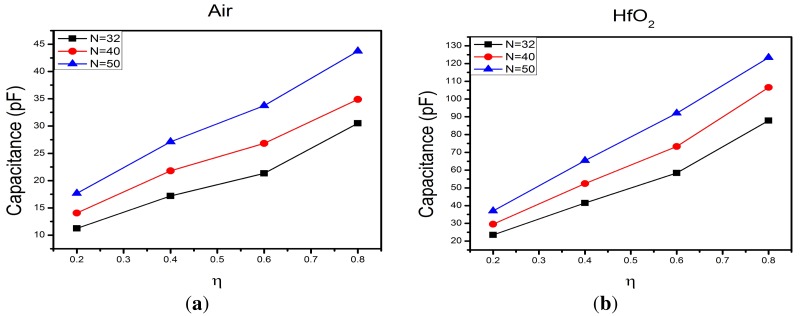
Numerical simulations for the capacitance for the IDCs for *N* = 32, *N* = 40 and *N* = 50 without and with hafnium coating. (**a**) Capacitance of the IDCs without hafnium coating; (**b**) Capacitance of the IDCs with hafnium coating.

The capacitance value of each sensor design can be calculated from COMSOL applications. The calculation of *C* is obtained by using the following formula

(2) $$C=\frac{2W_{e}}{\Delta V^{2}}$$

where *W_e_* is the stored electric energy and Δ*V* is the voltage across the IDC. The software calculates *W_e_* in the Electrostatic application mode by using the following formula

(3) $$W_{e}=\int_{\Omega }\left(D\cdot E\right)d\Omega$$

where *D* is the electric displacement, *E* is the electrostatic field and Ω is the domain of integration. The calculated capacitances as a function of *η* obtained from simulation are shown in Figure 4 for the IDCs with and without Hafnium.

From Figure 4 one can easily see that the highest capacitance is given for the IDCs with hafnium coating and that the capacitance increases with the number of electrodes *N*.

## 4. Fabrication Process

**Figure 5.**
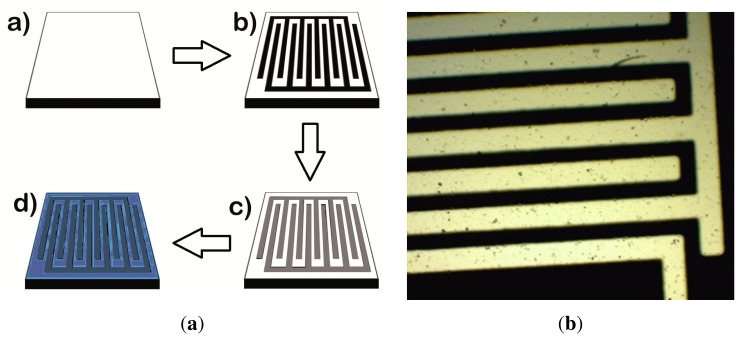
The figure shows on the left the fabrication process of the IDC, including (**a**) the silicon substrate; (**b**) photolithography; (**c**) evaporation of metal (Ni) and (**d**) HfO_2_ deposition, and on the right the final IDC device.

The initial design of the IDC was done using the AUTOCAD software [12]. The final design of the IDC from AUTOCAD was printed in negative and then was transferred into a film. The bottom of the IDC was deposited on Si substrate using photolithography and e-beam evaporation techniques. The hafnium oxide layer was grown on the IDC-coated substrate by atomic layer deposition in a Savannah 100 system by Cambridge Nanotech using tetrakis(dimethylamido)hafnium(IV) (TDMAHf) as precursor and deionized water as reactant. Five hundred cycles corresponding to approximately 62 nm thickness of HfO_2_ were performed. Figure 5 shows a representation of the fabrication process of the IDCs and one of the fabricated IDCs using photolithography and etching techniques on a Si substrate.

## 5. Experimental Results

We fabricated three nickel IDCs on a silicon substrate with the following values given in Table 1.

The impedance of the IDCs with and without HfO_2_ was measured as a function of frequency. A vector impedance analyzer (VIA) was used, with a frequency range of 2 MHz to 1 GHz, to measure the IDCs. The data from the impedance analyzer can be used in the following equation to obtain the total capacitance

(4) $$Z=\sqrt{R^{2}+{\left(\frac{1}{\omega C}\right)}^{2}}$$

**Figure 6.**
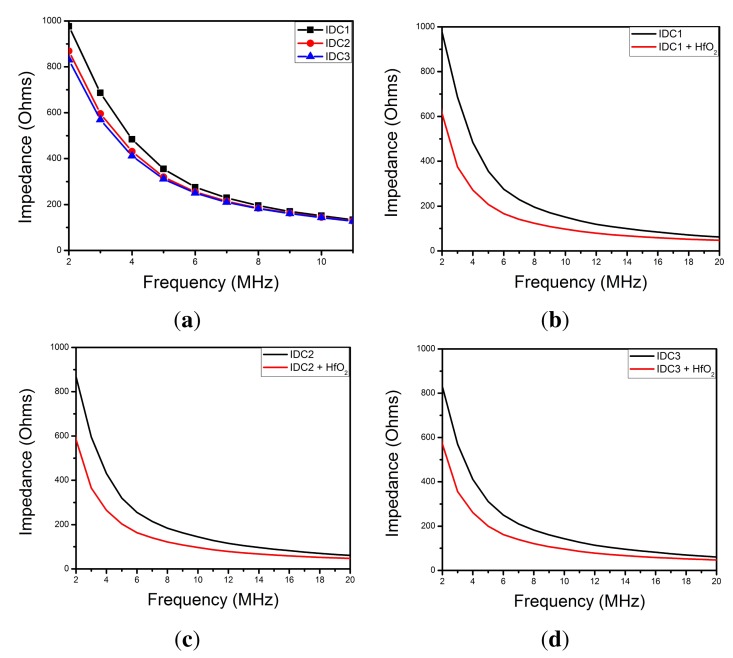
Plots showing the impedance for the following values: (**a**) *N* = 32, *N* = 40 and *N* = 50, (**b**) IDC1 with and without HfO_2_, (**c**) IDC2 with and without HfO_2_ and (**d**) IDC1 with and without HfO_2_.

Note that if the capacitance increases then the impedance decreases. In Figure 6 we show the impedance as a function of the number of electrodes and we also show the change in impedance after the IDCs are coated with HfO_2_. It can be seen from the plots that the impedance decreases at greater number of electrodes and after the IDCs are coated with HfO_2_, as expected from the numerical simulations.

**Figure 7.**
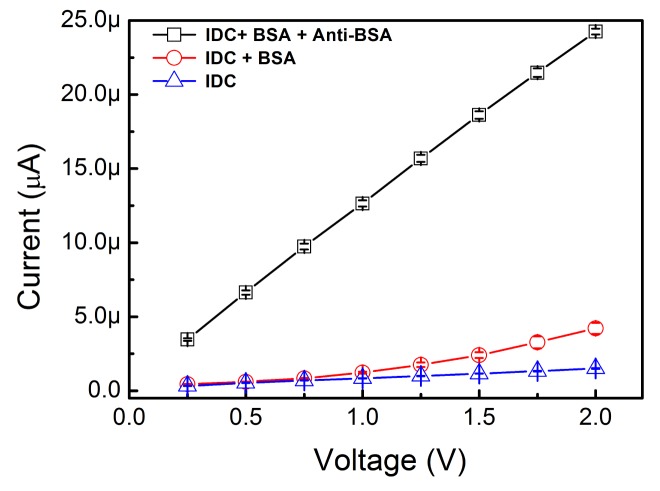
The current *vs.* voltage measurements of the IDC sensor with *N* = 40 incorporating Bovine Serum Albumin (BSA) and Anti-Bovine Serum Albumin (Anti-BSA). Note the increase in the conductivity when adding BSA or Anti-BSA to the sensor.

To validate the IDC sensor experimentally, we present an application of the IDC sensor with *N* = 40 as a proof-of-concept. We recorded the current *vs.* voltage curves for the IDC with *N* = 40 to evaluate the effect of incorporating Bovine Serum Albumin (BSA) and Anti-Bovine Serum Albumin (Anti-BSA) on the surface of the electrode. Figure 7 shows the current vs. voltage measurements of the IDC sensor. These measurements indicate that the BSA and Anti-BSA concentration influences the electrical characteristics of the deposited samples. An increase in conductivity was observed when adding the BSA and a bigger increase when adding the Anti-BSA. These results show that our designed IDCs have potential to be used as immunosensors for disease diagnosis.

## 6. Conclusions

We have proven that the nickel IDC sensor coated with a 60 nm layer of hafnium dioxide increases the electrical response of the capacitance. The sensing hafnium dioxide layer has been tested for three different IDC sensor configurations for frequencies from 2 MHz to 20 MHz at room temperature. The obtained results have been compared with the numerical simulation of the IDC sensor by COMSOL Multiphysics. Our results show that IDC sensors can be successfully designed using Finite Element Method software such as COMSOL. The performance of our IDC sensor was tested with Bovine Serum Albumin and Anti-Bovine Serum Albumin as a proof-of-concept that the IDC can be used as a sensor.

## Figures and Tables

**Table 1.** Fabricated interdigital capacitors.

**IDC**	**N**	**W** [*μ***m**]	**G** [*μ***m**]	**L** [**mm**]	*λ* [*μ***m**]
1	32	100	100	8	400
2	40	100	75	8	350
3	50	100	50	8	300

## References

[1] Mamishev A.V., Sundara-Rajan K., Yang F., Du Y., Zahn M. (2004). Interdigital sensors and transducers. IEEE Proc..

[2] Mukhopadhyay S.C. (2005). Novel planar electromagnetic sensors: Modeling and performance evaluation. Sensors.

[3] Boutejdar A., Abdel-Rahman A., Batmanov A., Burte P., Omar A. (2010). Miniaturized band-stop filter based on multilayer-technique and new coupled octagonal defected ground structure with interdigital capacitor. Microw. Opt. Technol. Lett..

[4] Hobdell J. (1979). Optimization of interdigital capacitors. IEEE Trans. Microw. Theory Technol..

[5] Esfandiari R., Maki D., Siracusa M. (1983). Design of interdigital capacitors and their application to GaAs monolithic filters. IEEE Trans. Microw. Theory Technol..

[6] Jackson J.D. (1999). Classical Electrodynamics.

[7] Alley G.D. (1970). Interdigital capacitors and their application to lumped-element microwave integrated circuits. IEEE Trans. Microw. Theory Technol..

[8] Kollipara R.T., Mohammed A.S., Plant T.K., Tripathi V.K. (1991). Modeling and design of interdigital structures. IEEE Trans. Electron Devices.

[9] Wang Y., Chong N., Cheng Y.L., Chan H.L.W., Choy C.L. (2003). Dependence of capacitance on electrode configuration for ferroelectric films with interdigital electrodes. Microelectron. Eng..

[10] Roberson J. (2006). High dielectric constant gate oxides for metal oxide Si transistors. Rep. Prog. Phys..

[11] Igreja R., Dias C.J. (2004). Analytical evaluation of the interdigital electrodes capacitance for a multi-layered structure. Sens. Actuators A Phys..

[12] Gevorgian S.S., Martinsson T., Linner P.L.J., Kollberg E.L. (1996). CAD Models for multilayered structure interdigital capacitors. IEEE Trans. Microw. Theory Technol..

